# The Oncogenic Role and Immune Infiltration for CARM1 Identified by Pancancer Analysis

**DOI:** 10.1155/2021/2986444

**Published:** 2021-10-27

**Authors:** Kui Liu, Jing Ma, Jiao Ao, Lili Mu, Yixian Wang, Yue Qian, Jin Xue, Wei Zhang

**Affiliations:** ^1^Department of Nephrology, Air Force Hospital of Western Theater Command, Chengdu, China; ^2^Department of Nursing, Guizhou Nursing Vocational College, Guiyang, China; ^3^Department of Pathogen Biology, Guizhou Nursing Vocational College, Guiyang, China; ^4^Department of Basic Medicine, Guizhou Nursing Vocational College, Guiyang, China

## Abstract

Chromatin-modifying enzymes, especially protein arginine methyltransferases (PRMTs), have been identified as candidate targets for cancer. Cellular or animal-based evidence has suggested an association between coactivator-linked arginine methyltransferase 1 (CARM1) and cancer progression. However, the relationship between CARM1 and patient prognosis and immune infiltration in pancancer patients is unknown. On the basis of the GEO and TCGA databases, we first investigated the possible oncogenic functions of CARM1 in thirty-three tumor types. CARM1 expression was elevated in many types of tumors. In addition, there was a significant association between CARM1 expression and the survival rate of tumor patients. Uterine corpus endometrial carcinoma (UCES) samples had the highest CARM1 mutation frequency of all cancer types. In head and neck squamous cell carcinoma (HNSC) and lung squamous cell carcinoma (LUSC), CARM1 expression was associated with the level of CD8+ T cell infiltration, and cancer-associated fibroblast infiltration was also observed in other tumors including kidney renal papillary cell carcinoma (KIRC) and prostate adenocarcinoma (PRAD). CARM1 was involved in immune modulation and played an important role in the tumor microenvironment (TME). Furthermore, activities associated with RNA transport and its metabolism were included in the possible mechanisms of CARM1. Herein, our first pancancer research explores the oncogenic role of CARM1 in various tumors. CARM1 is associated with immune infiltrates and can be employed as a predictive biomarker in pancancer.

## 1. Introduction

The incidence rate of cancer has risen significantly and has become one of the leading causes of death in humans over the last few years [1]. With significant knowledge of the mechanisms underlying cancer development and progression, several approaches to combating cancer have been devised [2]. By assuming the complexities of oncogenesis, it is critical to use pancancer expression analysis for identifying the similarities and differences between the gene of interest and cellular alterations that occur during cancer progression, as well as their association with the clinical prognosis. The publicly evadible TCGA project and GEO databases both comprise gene function datasets from various cancers, allowing for pancancer research [3, 4].

Posttranslational modification of the protein, including phosphorylation, methylation, and ubiquitination, is a covalent modification of protein catalyzed by enzymes, which can occur on histones and nonhistones and participate in many life processes. Methylation is an important posttranslational modification, while arginine is extremely susceptible to methylation due to its active biochemical properties. Protein arginine methyl transferase (PRMTS) is responsible for catalyzing this modification. According to recent research, abnormal arginine methylation modification is associated with the onset and progression of malignancies [5]. CARM1, coactivator-linked arginine methyltransferase 1, was first discovered due to its interaction with GRIP1 (p160 steroid receptor coactivator) [6, 7]. CARM1 regulates a wide range of biological activities, including DNA damage response, transcription, pre-mRNA splicing, protein stability regulation, and cell division. CARM1 methylates H3R17, H3R26, and H3R42, and the obtained H3R17/R26/R42ME2 markers are related to transcriptional activation [8]. Many nonhistone methylation substrates of CARM1 have been determined, such as CARM1 catalyzing BAF155 and PKM2 [9]. According to recent research, CARM1 enhances cancer progression and metastasis by catalyzing the methylation of tumor proteins which leads to an investigation of the molecular mechanism, revealing CARM1's correlation with cancer genesis and progression [10]. Furthermore, the current study summarized the existing cell- or animal-based data with the correlation between CARM1 and different cancers. However, despite a large body of clinical data, there is currently no pancancer research on the association between CARM1 and other cancer types.

In this study, the TCGA project and GEO databases were used for the first time to perform a pancancer review of CARM1 for evaluating the possible molecular mechanism of CARM1 in relation to cancer progression and clinical prognosis. Herein, we also added a set of parameters such as gene expression, cancer prognosis, genetic change, immunological infiltration, and the corresponding cellular pathway for investigating CARM1's possible molecular mechanism in the etiology or clinical prognosis of various cancers.

## 2. Materials and Methods

### 2.1. The Cancer Genome Atlas (TCGA)

TCGA is a freely accessible web-based cancer genomics database that contains large quantities of NGS data (with >11,000 tumors across 33 types of cancer until 2021). It provides datasets for the expression of genes, methylation, copy number alteration (CNA), and clinical information [11, 12].

### 2.2. Genotype-Tissue Expression (GTEx)

Using RNA sequencing, GTEx provides gene expression data from 53 healthy tissue sites in approximately 1000 individuals and is freely available to the public. UCSC Xena datahubs (http://xena.ucsc.edu/) were used to obtain RNA-seq data, such as GTEx and TCGA data, for a pancancer differential expression of CARM1. The integration of TCGA and GTEx was carried out by the limma package and R-version 3.6.3.

### 2.3. TISIDB

TISIDB (http://cis.hku.hk/TISIDB/in-dex.php) is an integrated database used for the interaction of tumor-immunity and genes [13]. TISIDB was employed for the CARM1 gene expression analysis of various molecular subtypes of tumor samples obtained from TCGA.

### 2.4. HPA

HPA (https://www.proteinatlas.org/) is a dataset that maps human proteins in organs, tissues, and cells through a combination of omics techniques [14, 15]. Herein, we employed the HPA program to show the distribution of CARM1 mRNA in healthy and cancerous tissues. Furthermore, in the pathology Atlas and tissue Atlas panels, we obtained immunohistochemistry images of CARM1 proteins.

### 2.5. cBioPortal

The cBioPortal for cancer genomics (http://www.cbioportal.org) is a database of cancer genomics. Using this portal, CARM1 mutations and CNA (copy number alterations) were observed in various cancer types.

### 2.6. Survival Analysis

The PrognoScan dataset (http://dna00.bio.kyutech.ac.jp/PrognoScan/index.html) aims to make meta-analyses of gene prognostic value easier by evaluating the correlation between gene expression and related outcomes in a variety of reported cancer microarray datasets [16]. In this view, we used PrognoScan to investigate the association between the expression of CARM1 and patient outcomes in various cohorts. We used the “survival” package (exist) in *R* to conduct survival analysis and calculated the log-rank *P* value and hazard ratio (HR) with 95% CI in the TCGA portal. Forest plots (created with R's “forest plot” package) and survival curves were used to show the results.

### 2.7. Immune Infiltration Analysis

TIMER2.0 comprehensive resource was employed to evaluate the association between the expression of CARM1 and immune infiltrates in all TCGA cancers. CD8+ T cells and fibroblasts (cancer-associated) were chosen. Immune infiltration was estimated using the TIMER2.0, CIBERSORT-ABS, XCELL, QUANTISEQ, EPIC algorithms, MCPCOUNTER, and CIBERSORT. Spearman's rank correlation test was employed for calculating the partial correlation (cor) and *P* values. A scatter plot and a heatmap were used to visualize the obtained results.

### 2.8. CARM1-Related Gene Enrichment Analysis

The STRING (https://string-db.org/) website was used to query the protein and organism name, i.e., CARM1 and *Homo sapiens*, respectively. The following key parameters were then set: minimum necessary interaction score (meaning of network edges (“evidence”), “Low confidence (0.150)”), active interaction sources (“experiments”), and the maximum number of interactors to display (“no more than 50 interactors”). The CARM1-interacting proteins that had been evaluated experimentally were then collected.

The “Similar Gene Detection” module of GEPIA2 was employed to get the highest; a hundred CARM1-associated targeting genes supported traditional tissues and the TCGA tumor datasets. Paired gene Pearson correlation analysis was performed for CARM1 and hand-picked genes by applying the “correlation analysis” module of GEPIA2. For the dot plot, the log2 TPM was used, followed by indicating the correlation coefficient (*R*) and the *P* value. We used TIMER2.0's “Gene Corr” module to provide heatmap information of the certain genes, including the cor and *P*-value by the analysis of Spearman rank correlation. Furthermore, we have the potential to integrate the two sets of information for conducting the KEGG pathway analysis (Kyoto Encyclopedia of Genes and Genomes).

Briefly, the cistron lists were uploaded into the DAVID database. This database is used for annotation, integrated discovery, and mental picture generation. The hand-picked symbols (“OFFICIAL GENE SYMBOL”) and species (“*Homo sapiens*”) were also uploaded with the cistron lists in order to obtain information from the accessible annotation map. Finally, the enriched pathways were interpreted using the *R* packages “tidyr” and “ggplot2.” In addition, we often used the *R* package, i.e., clusterProfiler to perform GO analysis. The cnetplot functions, such as node label, circular, and ColorEdge, shortly represented as *T*, F, and *T*, accordingly, were used. The data for biological process (BP), molecular function (MF), and cellular component (CC) were shown as cnetplots. In the current research, the *R* language programming code (R-3.6.3) was used (https://www.r-project.org/). The two-tailed *P* and lt, i.e., 0.05 was found to be statistically significant.

### 2.9. Statistical Analysis

The HPA site was used to determine the CARM1 in different carcinomas. The CARM1 expression in cancer was evaluated via Oncomine, matched GTEx, and TIMER2.0 databases. PrognoScan and the *R* package were used to plot the survival curves using data from the TCGA dataset. HR, 95% CI, and log-rank *P* values were used to represent the survival results. cBioPortal was used to examine the mutation and CNV profiles. The immune infiltration analysis was performed using the *R* package and the TIMER2.0 website. Student's *t*-test was employed to correlate two groups, while ANOVA was used for comparing multiple groups. Pearson's correlation analysis was used to determine the level of correlation between specific variables, with the R/rho variables 0–0.19, 0.20–0.39, 0.40–0.59, 0.60–0.79, and 0.80–1.00 denoting the correlation strength (very weak, weak, moderate, solid, and extremely strong). The *P* < 0.05 was considered statistically significant.

## 3. Results

### 3.1. Expression Levels of CARM1 in Human Normal Tissues

**Figure 1:**
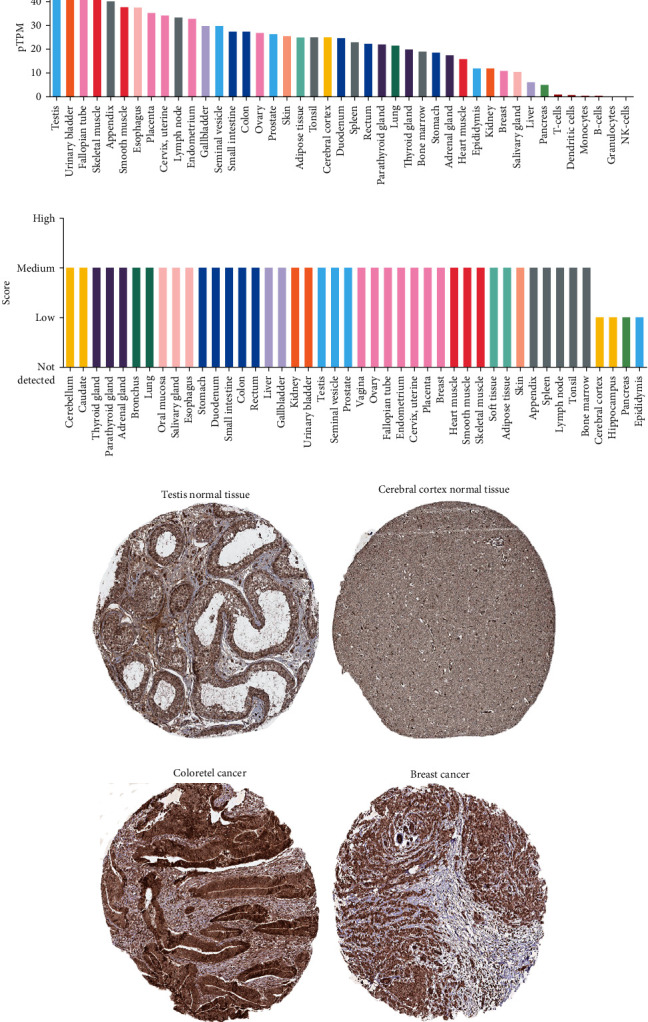
The expression of CARM1 in normal as well as cancer tissues of a human in the HPA database. (a) The expression of CARM1 in healthy tissues (human). (b) The expression of CARM1 protein in healthy tissues (human). (c–f) Characteristic IHC images of CARM1 expression in tissues, such as testis normal tissue, cerebral cortex normal tissue, colorectal cancer, and breast cancer tissues.

Using the HPA database to find CARM1 protein and mRNA expression profiles in human tissues, we investigated CARM1 expression in various tumor and normal tissues. The expression of CARM1 mRNA was found to be enriched in skeletal muscle and the urinary system, as shown in Figure 1(a). The expression of CARM1 protein was then evaluated and was found to be commonly distributed, albeit at elevated levels, in a variety of normal tissues, as shown in Figure 1(b). Immunohistochemistry (IHC) results revealed that CARM1 protein was mostly found in the nucleus and cytoplasm, with low expression in peritubular cells of normal testis tissues and neuronal cells of normal cerebral cortex tissues (Figures 1(c) and 1(d)). The obtained results also indicated an elevated expression of CARM1 in several cancers including colorectal and breast cancer, as shown in Figures 1(e) and 1(f). The IHC results are also given in Table 1.

### 3.2. The Expression of CARM1 mRNA in Human Cancers

**Figure 2:**
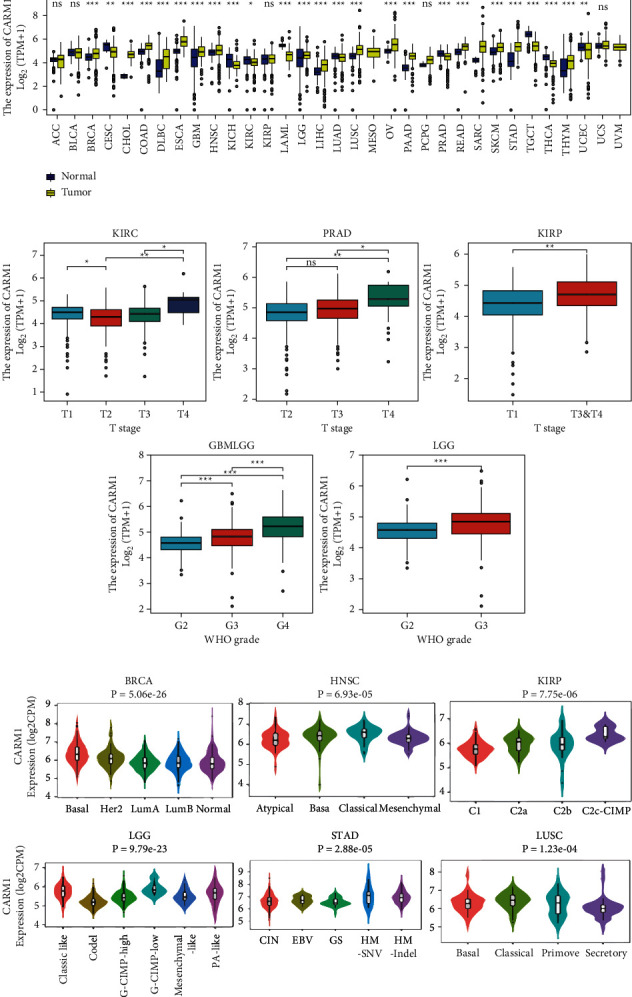
The mRNA levels of CARM1 in human cancers. (a) Tissues from TCGA and GTEx used for evaluating the mRNA expression of CARM1 between healthy and tumor tissues. (b) The link between the expression of CARM1 mRNA and various pathological phases in patients with various cancers from TCGA. (c) The CARM1 expression in various molecular subtypes of cancers via TISIDB (^*∗∗∗*^*P* value, ^*∗∗*^*P* value, ^*∗*^*P* value, and *P* value indicate ≤0.001, ≤0.01, ≤0.05, and ≤0.1, accordingly).

To evaluate the characteristics of CARM1 mRNA expression, we combined tumor and normal samples from TCGA and the GTEx databases, respectively. Collectively, the expression of CARM1 was elevated in various cancers including breast invasive carcinoma (BRCA), cholangiocarcinoma (CHOL), lymphoid neoplasm diffuse large B cell lymphoma (DLBC), colon adenocarcinoma (COAD), glioblastoma multiforme (GBM), esophageal carcinoma (ESCA), LGG (brain lower-grade glioma), HNSC, LUSC, OV (ovarian serous cystadenocarcinoma), rectum adenocarcinoma (READ), liver hepatocellular carcinoma (LIHC), stomach adenocarcinoma (STAD), pancreatic adenocarcinoma (PAAD), and thymoma (THYM), as shown in Figure 2(a). When tumors and their matched adjacent tissues were involved in the TCGA database, the expression of CARM1 was elevated in BRCA, bladder urothelial carcinoma (BLCA), COAD, CHOL, HNSC, ESCA, lung adenocarcinoma (LUAD), LIHC, STAD, and LUSC, while its decreased expression was also observed in KIRC and kidney chromophobe (KICH) via the TIMER database (Supplementary Figures S1a and S1b).

The mRNA expression patterns of CARM1 were evaluated in many clinical phases and molecular subtypes. Significant variations were observed in the expression of CARM1 in various clinical phases of KIRC, PRAD, kidney renal papillary cell carcinoma (KIRP), LGG, and glioma (GBMLGG), as shown in Figure 2(b). The expression of CARM1 considerably varied in various molecular subtypes of HNSC, BRCA, KIRP, STAD, LGG, and LUSC, as shown in Figure 2(c). In other types of cancers, no correlation was observed between the expression of CARM1 and cancer phase or molecular subtype (Supplementary Figures S1c-S1d).

### 3.3. Correlation Analysis between the Prognostic Value and CARM1 mRNA Expression

**Figure 3:**
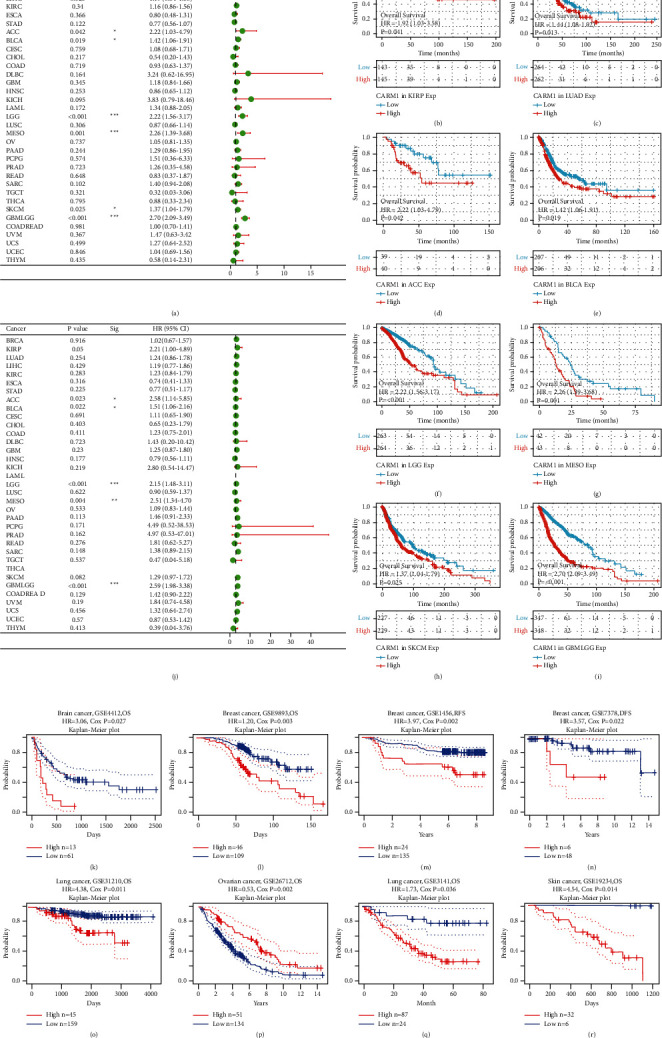
Survival analysis was employed to compare the elevated and lower expression of CARM1 in various kinds of cancer in the TCGA and GEO datasets. (a, j) The link between the expression of CARM1 and prognosis in patients (DSS and OS) of various cancers in the TCGA database (^*∗∗∗*^*P* < 0.001, ^*∗∗*^*P* < 0.01, and ^*∗*^*P* < 0.05). (b–i) OS curves having statistical significance in TCGA for eight cancer types (KIRP, LUAD, ACC, LGG, BLCA, MESO, GBMLGG, and SKCM). (k–r) Survival curves in eight cohorts GSE4412, GSE9893, GSE1456, GSE7378, GSE31210, GSE26712, GSE3141, and GSE1923) with significance.

Clinical data and TCGA RNA-seq (downloaded from UCSC Xena) were used to examine the prognosis of 33 TCGA cancer types to see if CARM1 has an effect on cancer patient prognosis. The obtained results revealed that elevated expression of CARM1 was considerably associated with bad overall survival (OS) in KIRP (HR = 1.92, 95% CI = 1.03–3.58, *P* = 0.041), LUAD (HR = 1.44, 95% CI = 1.08–1.92, *P* = 0.013), ACC (adrenocortical carcinoma; HR = 2.22, 95% CI = 1.03–4.79, *P* = 0.042), BLCA (HR = 1.42, 95% CI = 1.06–1.91, *P* = 0.019), LGG (HR = 2.22, 95% CI = 1.56–3.17, *P* < 0.001), MESO (mesothelioma; HR = 2.26, 95% CI = 1.39–3.68, *P* = 0.001), SKCM (skin cutaneous melanoma; HR = 1.37, 95% CI = 1.04–1.79, *P* = 0.025), and GBMLGG (HR = 2.70, 95% CI = 2.09–3.49, *P* < 0.001), as shown in Figure 3(a). The survival curves are shown in Figures 3(b)–3(i), in which *P* < 0.05 was considered as statistically significant. Disease-free survival (DSS) was also evaluated to prevent bias arising from noncancer cases, as shown in Figure 3(j).

To further examine the prognostic potential of CARM1, the PrognoScan database was employed to evaluate the correlation between CARM1 and the survival outcomes of patients suffering from cancer patients. The obtained results were according to eight cohorts (GSE4412 [17], GSE9893 [18], GSE1456 [19, 20], GSE7378 [21], GSE31210 [22], GSE26712 [23], GSE3141 [24], and GSE19234 [25, 26]), which indicated that an elevated expression of CARM1 was significantly associated with a bad prognosis (COX *P* < 0.05; Figures 3(k)–3(o), 3(q), and 3(r)). On the other hand, elevated expression of CARM1 was linked with the considerable rate of prognosis in ovarian cancer, as shown in Figure 3(p) (COX *P* = 0.002). Supplementary Table 1 contains detailed information on these cohorts.

### 3.4. Gene Expression and Mutation Analysis of CARM1 across Various Human Cancers

**Figure 4:**
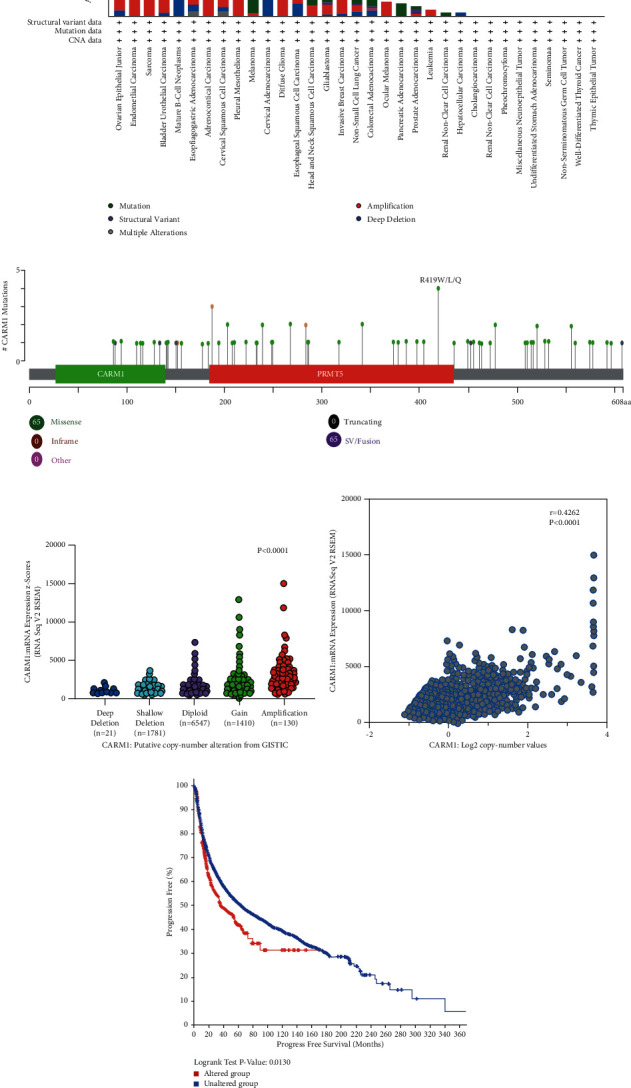
CARM1 mutation features in various TCGA tumors. cBioPortal tool was used to evaluate CARM1 mutation features for the TCGA tumors. The frequency of mutations is shown along with the mutation type (a) and mutation site (b). The link between CARM1 copy number and expression of mRNA indicated in the dot plot (c) and correlation plot (d) by cBioPortal (e). Using the cBioPortal method, we determined the considerable association between CARM1 mutation status and progression-free survival in various tumors.

Genetic and epigenetic variations significantly contribute to the regulation of cancer initiation and immune tolerance. cBioPortal was used to explore genetic variations of CARM1. The mutation frequency of CARM1 in the TCGA database was then examined using cBioPortal (10967 samples in 32 studies), and the obtained results revealed that OV and uterine corpus endometrial carcinoma (UCES) had a high mutation rate, with CARM1 mutations accounting for more than 7% of the total, as shown in Figure 4(a), followed by detecting 85 mutation sites (i.e., 65 missense, 7 truncating, and 13 fusion mutations) locating between amino acids 0 and 608, as shown in Figure 4(b). As shown in Figure 4(c), CARM1 deletion was found in more than one-third of human cancers, and different human cancers with CARM1 deletion consistently had lower expression of mRNA relative to those with diploid CARM1. Furthermore, in various TCGA human cancers, a positive association was found between CARM1 copy number and mRNA expression (Figure 4(d)). Different human cancer cases with altered CARM1 showed the poorer prognosis in progression-free survival (*P* = 0.013), but not overall survival (*P* = 0.311), disease-free (*P* = 0.103), and disease-specific (*P* = 0.0626) relative to cases without CARM1 variations (Figure 4(e), Supplementary Figure S2).

### 3.5. Association between mRNA Expression of CARM1 and the Immune Infiltration

**Figure 5:**
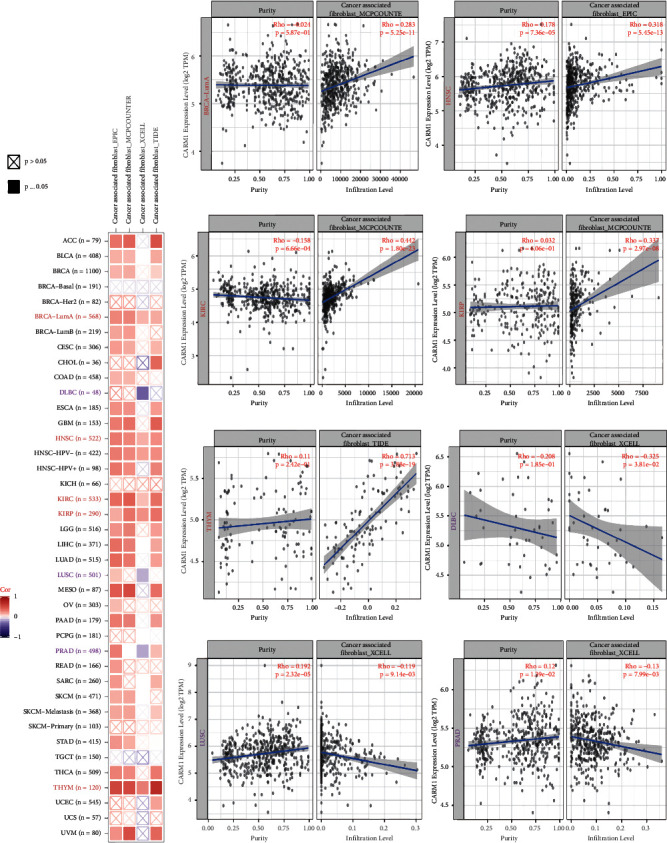
Correlation investigation of CARM1 expression and immune infiltration of fibroblasts associated with cancer. (a) The possible association between CARM1 expression and the infiltration level of fibroblasts (cancer-linked) investigated using various algorithms across all cancer types in the TCGA. (b) Significant correlations of CARM1 expression with the infiltration level of fibroblasts (cancer-linked) in KIRC, THYM, BRCALumA, HNSC, KIRP,. PRAD, DLBC, and LUSC by using a single algorithm.

Tumor-infiltrating immune cells (TIICs) were significantly associated with the development or metastasis of cancer [27, 28]. The involvement of several TIICs in the stroma of TME has been confirmed to be modulated by fibroblasts (cancer-linked) [29]. We, therefore, investigated the possible association between the infiltration level of various immune cells and the expression of the CARM1 gene in TCGA cancer types using the CIBERSORT, TIMER, QUANTISEQ, CIBERSORT-ABS, MCPCOUNTER, EPIC, and XCELL algorithms. Based on the data obtained from algorithms, we found a statistically negative association between immune infiltration of CD8+ T cells and the expression of CARM1 in HNSC, LUSC, and SKCM-metastatic tumors after a series of analyses, as shown in Figure S3. Furthermore, for the TCGA tumors of KIRC, THYM, BRCALumA, HNSC, and KIRP, we found a statistically positive association between the expression of CARM1 and the approximate infiltration value of fibroblasts (cancer-linked), but a negative correlation was observed for PRAD, DLBC, and LUSC, as shown in Figure 5(a). Meanwhile, Figure 5(b) shows the scatterplot data of the underlined tumors developed using a single algorithm. For instance, the degree of CARM1 expression in PRAD was found to be negatively linked with the immune infiltration level of fibroblasts (cancer-linked) using the XCELL algorithm (Rho = −0.13, *P* = 7.99*e* − 03).

### 3.6. Enrichment Analysis of CARM1-Associated Partners in Pancancer

**Figure 6:**
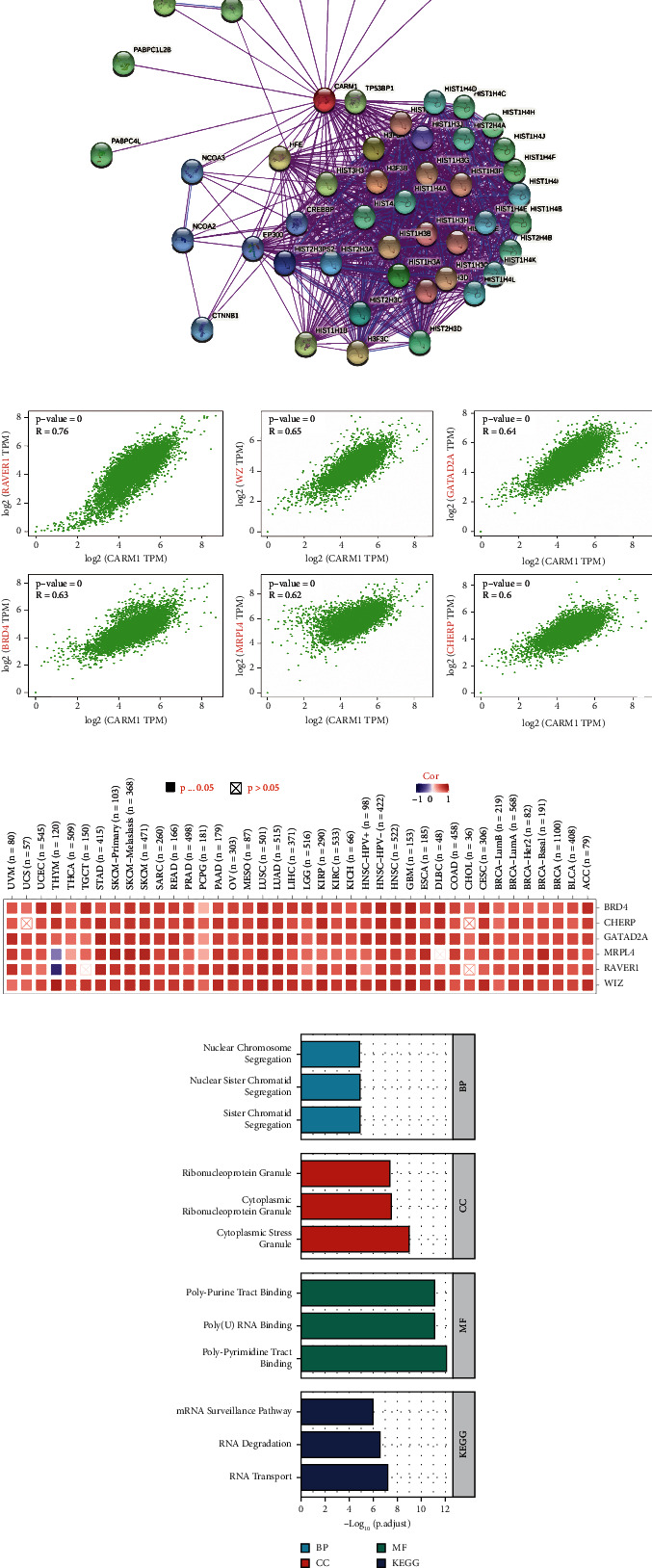
CARM1-associated gene enrichment analysis. (a) The STRING database used to obtain CARM1-binding proteins that were evaluated experimentally. (b) The GEPIA2 approach used to find the top 100 CARM1-related genes in the TCGA data, and the expression correlation between CARM1 and chosen target genes including RAVER1, WIZ, GATAD2A, BRD4, MRPL4, and CHERP was examined. (c) In the detailed cancer types, the equivalent heatmap data are displayed. (d) GO/KEGG pathway analysis conducted according to the CARM1-binding and interacted genes.

The targeting CARM1-interacting proteins and genes linked to CARM1 expression were evaluated for many pathway enrichment studies to determine the CARM1 gene's molecular mechanism in cancer genesis and progression. The STRING tool identified a total of 50 CARM1-interacting proteins, which were confirmed by experimental data. The interaction network of these proteins is shown in Figure 6(a). The GEPIA2 was used to integrate all of the top 100 genes, and TCGA tumor expression data were identified that were linked with the expression of CARM1. As shown in Figure 6(b), the expression of CARM1 was positively linked with that of RAVER1 (ribonucleoprotein, PTB binding 1) (*R* = 0.76), WIZ (WIZ zinc finger) (*R* = 0.65), GATAD2A (GATA zinc finger domain containing 2A) (*R* = 0.64), BRD4 (bromodomain containing 4) (*R* = 0.63), MRPL4 (mitochondrial ribosomal protein L4) (*R* = 0.62), and CHERP (calcium homeostasis endoplasmic reticulum protein) (*R* = 0.60) genes (all *P* < 0.001). In many forms of cancer, the heatmap data revealed a positive link between CARM1 and the underlined five genes (Figure 6(c)). To conduct KEGG and GO enrichment analyses, we merged the two datasets. According to the KEGG data in Figure 6(d), “RNA transport” and “mRNA surveillance cascade” might be associated with the impact of CARM1 on cancer pathogenesis. The GO enrichment analysis' findings have revealed that many of the underlined genes are associated with the metabolism cascades or cellular biology of RNA including nuclear chromosome segregation, ribonucleoprotein granule, sister chromatid segregation, polypurine tract binding, polypyrimidine tract binding, and others (Figure 6(d)).

## 4. Discussion

CARM1, also known as PRMT4, is the first of the PRMT protein family members to be associated with transcriptional activity [30]. CARM1 acts through methylated histones, transcription factors, coregulators, splicing factors, and RNA polymerase I to regulate a variety of cellular functions, including DNA damage repair, mRNA splicing, and cell cycle progression. CARM1 is recruited at the E2F1 promoter in breast cancer, which upregulates E2F1 expression and alters the expression of downstream cell cycle-related proteins, promoting the proliferation of breast cancer cells [31]. Moreover, CARMI is highly correlated with survival and involved in tumor invasion and metastasis in various diseases such as prostate cancer [32], making it crucial in disease research and drug development. Recent studies have found that inhibition of CARM1 in both tumor cells and T cells produces beneficial antitumor effects. Inhibition of CARM1 in T cells increased the activity of killer T cells and promoted the formation of memory T cells, while inhibition of CARM1 in tumor cells promoted the expression of interferon-stimulated genes (ISGs), induced dsDNA breaks in tumor cells, and ultimately activated the cGAS-STING pathway to inhibit tumor cell growth [33]. CARM1 knockout mice were born dead and showed deficiency in T cell development and cell differentiation [34], indicating that CARM1 has a very important role at both the cellular and individual levels. As a result, CARM1 could be employed as a new anticancer immunotherapy drug target or in combination with other immune checkpoint inhibitors to increase immune responses and infiltration in cancers. The findings suggested that CARM1 could be a potential target for anticancer immunotherapy. Combining anti-PD-L1 drugs with CARM1 depletion could also be used as a novel anticancer strategy.

We used the TCGA, TIMER, and GTEx databases to determine the level of CARM1 expression in cancers and normal tissues in the first stage of our study. Except for KIRC and KICH, CARM1 was shown to be substantially more expressed in most cancer types, which was consistent with the previous study in lung and colorectal cancers [10, 35]. These findings suggested that CARM1 does increase tumor growth and oncogenesis in human cancers.

Following that, the relationship between the prognosis and CARM1 expression was then explored. High expression of CARM1 was linked to a poor prognosis in a variety of cancer types, including KIRP, LUAD, ACC, BLCA, LGG, and SKCM, demonstrating CARM1 as a possible and effective prognostic marker for pancancer. Then, to evaluate CARM1's probable mechanisms of action, we looked at its expression in distinct molecular subtypes of malignant tumors. CARM1 expression was significantly varied in distinct molecular subtypes in most cancer types, suggesting that CARM1 might be a promising diagnostic pancancer biomarker as suggested by these findings. Furthermore, we found that CARM1 expression differed significantly amongst clinical subgroups of PRAD patients. CARM1 differential expression was seen in all gliomas with distinct clinical features, suggesting that CARM1 may play a role in cancer growth and progression.

Cancer growth and immune tolerance have also been influenced by genetic and epigenetic variations. For instance, mutant PD-L1 with structural changes results in abnormal expression of immunosuppression and PD-L1 [36]. An amplification of JAK2/PD-L1/PD-L2 (9p24.1) can result in a constitutively elevated expression of PD-L1 and a considerable reaction to checkpoint inhibitors [37]. Various mechanisms for controlling PD-L1 expression could represent the different roles of PD-L1 in many cellular localizations and cell types [38]. Preliminary analysis revealed that genetic, as well as epigenetic regulation of CARM1 expression, was carried out through CNA. GO/KEGG suggested that CARM1 was linked with oncogenic cascades (e.g., RNA degradation and mRNA surveillance cascades), as CARM1 has a significant role in immune regulation. The role of CARM1 as an oncogene and its exact mechanisms has not been fully understood.

The experimental validation and the public data analysis were performed using RT-qPCR and IHC differed in several ways. The current study focused on CARM1 expression in the cytoplasmic portion of cancer cells and assessing the complex TME via tissue microarray. We could not rule out the possibility of CARM1 expression in both the nucleus and the cytoplasmic portion of cancer cells. The variations of CARM1 expression in T lymphocytes and tumor cells are still unknown [39, 40]. The distribution of immune cells in the peripheral regions, tumor stroma, and the central core are strongly linked to the different immune infiltration patterns in the same tissue [41–43]. According to our findings, CARM1 displayed a significant association with tumor-infiltrating lymphocytes and played an important role in the TME. CARM1 expression, for example, was found to be substantially correlated with cancer-associated fibroblasts and CD8+ T cells. To perform precise and considerable evaluations, a large number of samples and in-depth analysis were needed in specific subgroups. Furthermore, the underlined data were according to the NGS (high-throughput sequencing technology) analysis derived from bulk cells and limited our evaluations.

In brief, we used integrated bioinformatics approaches, and the obtained results revealed that CARM1 expression may enhance immune infiltration and influence the survival rate of patients in pancancer, suggesting that CARM1 can be used as a prognostic biomarker and may provide knowledge to explore the malignancies and their pathologic processes of those prevalent cancers. It has also been suggested that CARM1 was considerably associated with multiple immune responses and infiltration, and immunotherapies along with CARM1 inhibitors may be an effective strategy to suppress the human ungracious tumors, mainly gliomas.

## Figures and Tables

**Table 1:** Clinical data and relative scores of IHC results from the HPA database.

Protein	Tissue	Histological type	Age	Gender	Location	Quantity	Intensity	Relative IHC sore
CARM1	Testis	Normal tissue	25	Male	Cytoplasmic/nuclear	<25%	Weak	1
CARM1	Cerebral cortex	Normal tissue	64	Female	Cytoplasmic/nuclear	<25%	Weak	1
CARM1	Colorectal cancer	Adenocarcinoma	56	Male	Cytoplasmic/nuclear	>75%	Moderate	6
CARM1	Breast cancer	Duct carcinoma	40	Female	Cytoplasmic/nuclear	>75%	Moderate	6

## Data Availability

All data generated or analyzed during this study are included within this article and its supplementary information files.

## Supplementary Materials

Supplementary Materials Figure S1. The transcription levels of CARM1 in human cancers. (a) The mRNA expression of CARM1 between tumor and their matched adjacent tissues was assessed using tissues from the TCGA database. (b) The mRNA expressions of CARM1 between tumor and normal tissues in multiple cancers via the TIMER database. (c) No difference was found between CARM1 expression and stages in these cancers. (d) No difference was found between CARM1 expression and molecular subtypes via TISIDB. Figure S2. The potential correlation between CARM1 mutation status and survival analysis of different tumors using the cBioPortal tool. (a) The potential correlation between CARM1 mutation status and disease-specific survival of different tumors using the cBioPortal tool. (b) The potential correlation between CARM1 mutation status and disease-free survival of different tumors using the cBioPortal tool. (c) The potential correlation between CARM1 mutation status and overall survival of different tumors using the cBioPortal tool. Figure S3. Correlation analysis between CARM1 expression and immune infiltration of CD8+ T cells. The negative correlation of CARM1 expression with the infiltration level of CD8+ T cells in HNSC, LUSC, and SKCM-metastases. Supplementary Table 1. CARM1 correlates with immune infiltration and impacts patient prognosis: a pancancer analysis. .
