# Supplementary material for: The Oncogenic Role and Immune Infiltration for CARM1 Identified by Pancancer Analysis
Source: J Oncol. 2021 Oct 27;2021:2986444. doi: 10.1155/2021/2986444 (PMC8566078; doi:10.1155/2021/2986444)
Supplement: Supplementary Materials — Figure S1. The transcription levels of CARM1 in human cancers. (a) The mRNA expression of CARM1 between tumor and their matched adjacent tissues was assessed using tissues from the TCGA database. (b) The mRNA expressions of CARM1 between tumor and normal tissues in multiple cancers via the TIMER database. (c) No difference was found between CARM1 expression and stages in these cancers. (d) No difference was found between CARM1 expression and molecular subtypes via TISIDB. Figure S2. The potential correlation between CARM1 mutation status and survival analysis of different tumors using the cBioPortal tool. (a) The potential correlation between CARM1 mutation status and disease-specific survival of different tumors using the cBioPortal tool. (b) The potential correlation between CARM1 mutation status and disease-free survival of different tumors using the cBioPortal tool. (c) The potential correlation between CARM1 mutation status and overall survival of different tumors using the cBioPortal tool. Figure S3. Correlation analysis between CARM1 expression and immune infiltration of CD8+ T cells. The negative correlation of CARM1 expression with the infiltration level of CD8+ T cells in HNSC, LUSC, and SKCM-metastases. Supplementary Table 1. CARM1 correlates with immune infiltration and impacts patient prognosis: a pancancer analysis. . [file 2986444.f1.docx]

**The oncogenic role and immune infiltration for CARM1 identified**

**by pan-cancer analysis**

Liu Kui^1^, Ma Jing^2^, Ao Jiao^2^, Lili Mu^3.4^, Yixian Wang^3.4^, Qian Yue^3.4^, Jin Xue^4^, Zhang Wei^3.4*^

1. Department of Nephrology, Air Force Hospital of Western Theater Command, Chengdu, China

2. Department of Nursing , Guizhou Nursing Vocational College, Guiyang, China

3. Department of Pathogen Biology, Guizhou Nursing Vocational College, Guiyang, China

4. Department of Basic Medicine ,Guizhou Nursing Vocational College,Guiyang,China

Correspondence to: Zhang Wei. Department of Pathogen Biology,Guizhou Nursing Vocational College, Guiyang, Guizhou 550000, China

Email:lawrence2013@163.com

**This file includes:** Supplementary Figures S1 to S3

Supplementary Table 1

**Figure S1** **The transcription levels of CARM1 in human cancers.**

1. The mRNA expression of CARM1 between tumor and their matched adjacent tissues was assessed using tissues from the TCGA database. **(b)** The mRNA expressions of CARM1 between tumor and normal tissues in multiple cancers via the TIMER database. **(c)** No difference was found between CARM1 expression and stages in these cancers. **(d)** No difference was found between CARM1 expression and molecular subtypes via TISIDB.

**Figure S2 The potential correlation between CARM1 mutation status and survival analysis of different tumors using the cBioPortal tool.**

1. The potential correlation between CARM1 mutation status and disease-specific survival of different tumors using the cBioPortal tool. **(b)** The potential correlation between CARM1 mutation status and disease-free survival of different tumors using the cBioPortal tool. **(c)** The potential correlation between CARM1 mutation status and overall survival of different tumors using the cBioPortal tool.

**Figure S3 Correlation analysis between CARM1 expression and immune infiltration of CD8+ T-cells.** The negative correlation of CARM1 expression with the infiltration level of CD8+ T-cells in HNSC, LUSC, and SKCM-Metastasis.

**Figure S1**


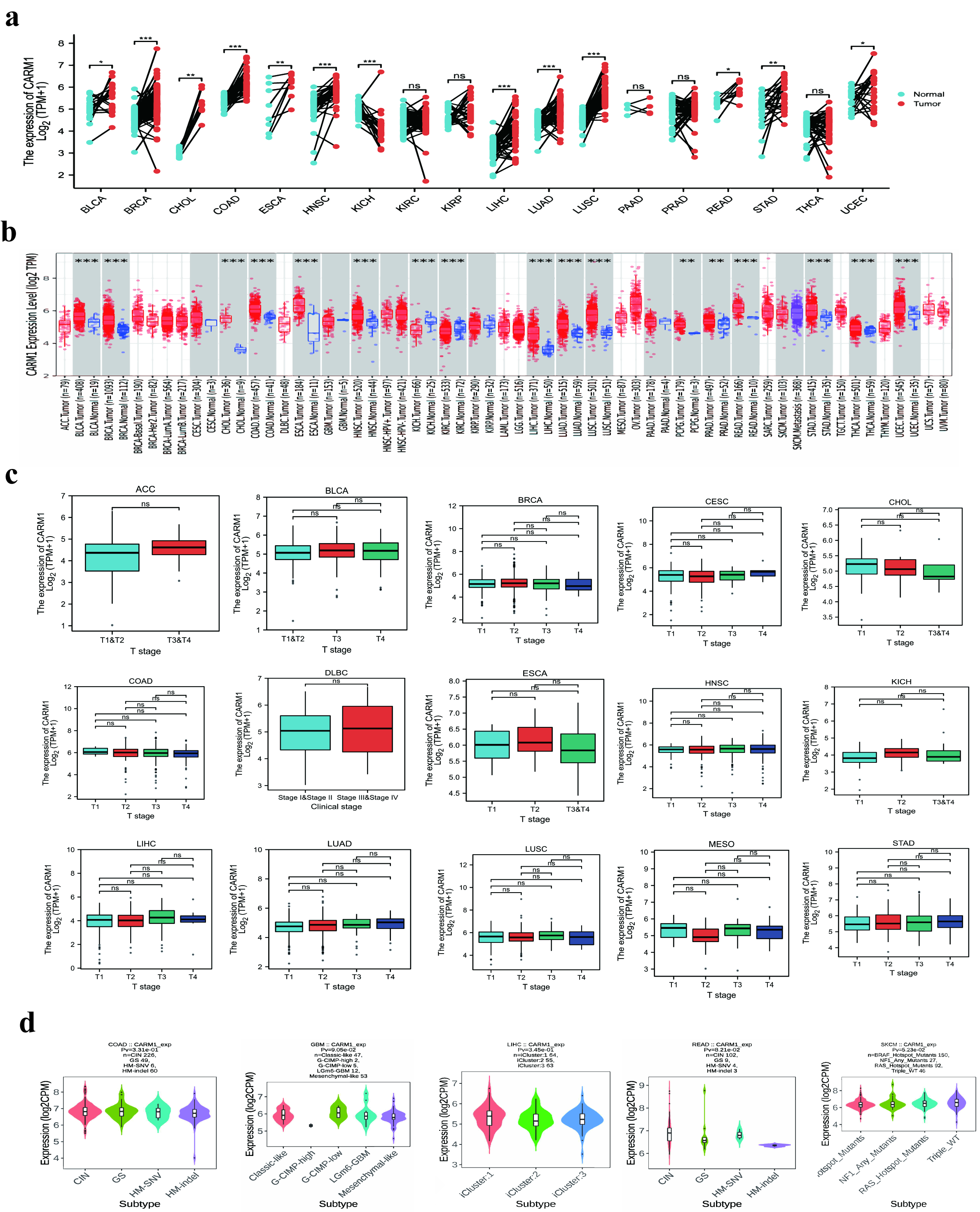


**Figure S2**

**
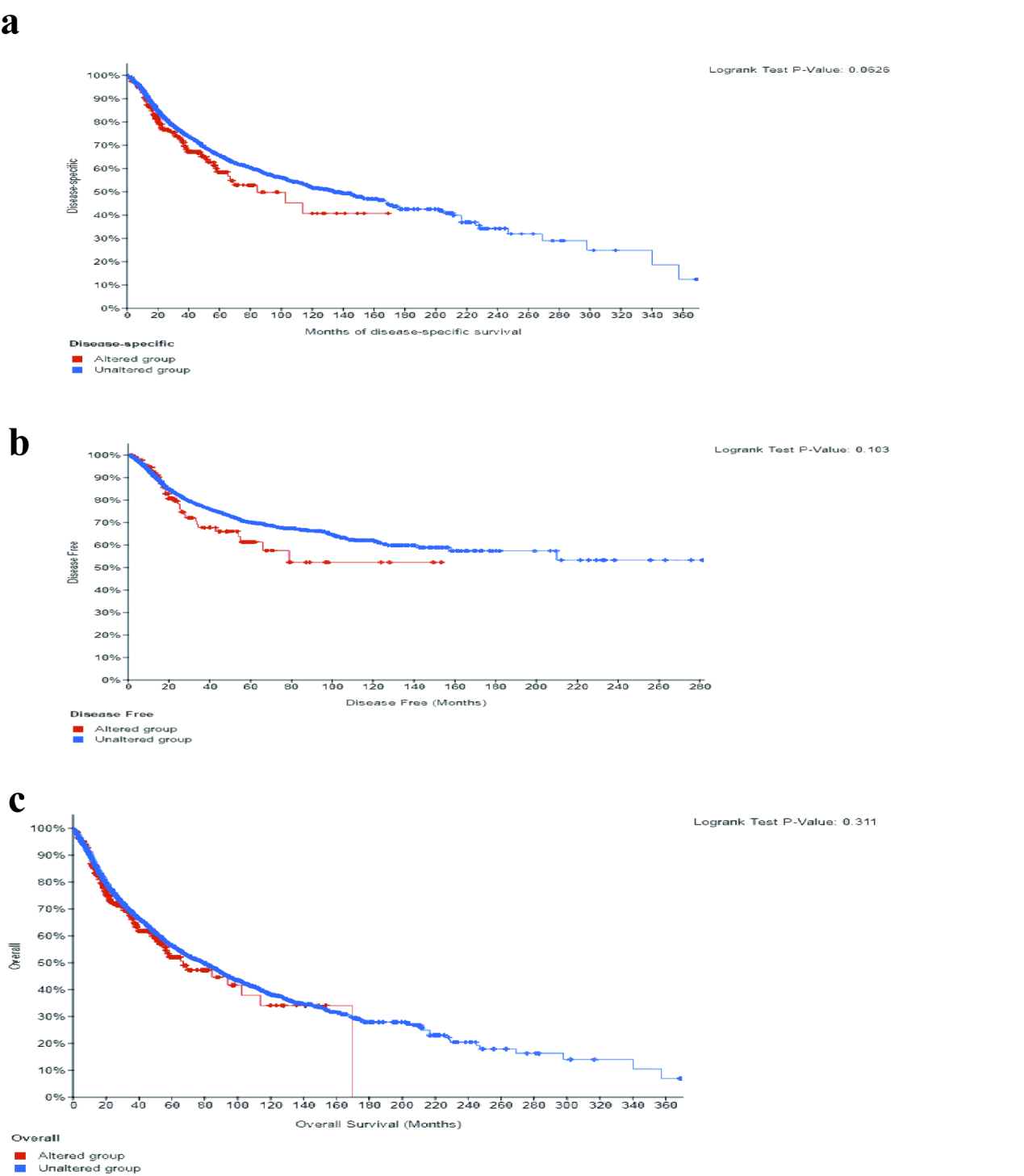
**

**Figure S3**

**
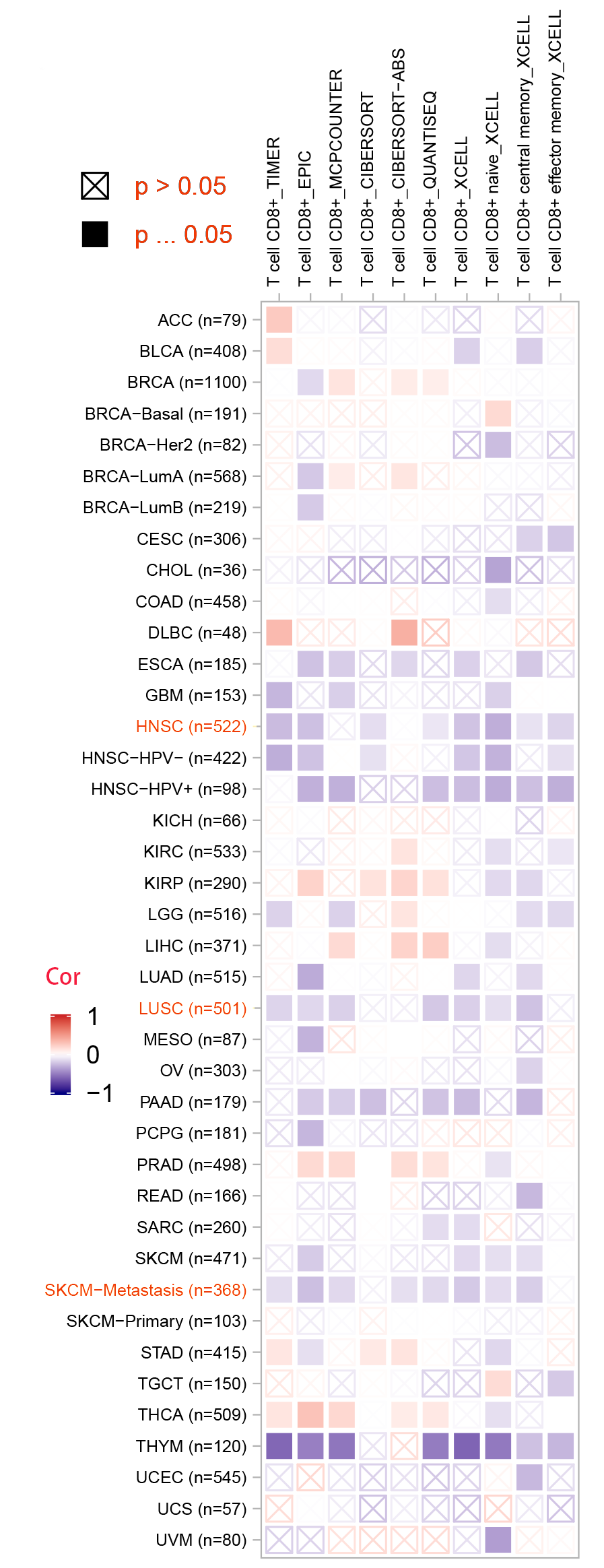
**

**Supplementary Table 1**

**CARM1 Correlates With Immune Infiltration and Impacts Patient Prognosis_ A Pan-Cancer Analysis**

| ID_NAME | DATASET | CANCER TYPE | SUBTYPE | ENDPOINT | N | CUTPOINT | HR  [95% CI-lowCI-upp] | COX *P*-VALUE | Sig |
| --- | --- | --- | --- | --- | --- | --- | --- | --- | --- |
| CARM1 | GSE4412 | Brain cancer | Glioma | Overall Survival | 74 | 0.824324 | 3.06 [1.13 - 8.29] | 0.0278428 | * |
| CARM1 | GSE9893 | Breast cancer |  | Overall Survival | 155 | 0.703226 | 1.20 [1.06 - 1.36] | 0.0034467 | ** |
| CARM1 | GSE1456 | Breast cancer |  | Relapse Free Survival | 159 | 0.767296 | 3.79 [1.61 - 8.89] | 0.00223335 | ** |
| CARM1 | GSE7378 | Breast cancer |  | Disease Free Survival | 54 | 0.888889 | 3.57 [1.19 - 10.67] | 0.0226827 | * |
| CARM1 | GSE31210 | Lung cancer | Adenocarcinoma | Overall Survival | 204 | 0.779412 | 4.38 [1.40 - 13.77] | 0.0113627 | * |
| CARM1 | GSE26712 | Ovarian cancer |  | Overall Survival | 185 | 0.724324 | 0.53 [0.36 - 0.80] | 0.00248741 | ** |
| CARM1 | GSE3141 | Lung cancer | NSCLC | Overall Survival | 111 | 0.216216 | 1.73 [1.03 - 2.90] | 0.0366964 | * |
| CARM1 | GSE19234 | Skin cancer | Melanoma | Overall Survival | 38 | 0.157895 | 4.54 [1.35 - 15.29] | 0.0144628 | ** |
